# Commensal Bacteria: An Emerging Player in Defense Against Respiratory Pathogens

**DOI:** 10.3389/fimmu.2019.01203

**Published:** 2019-05-31

**Authors:** Rabia Khan, Fernanda Cristina Petersen, Sudhanshu Shekhar

**Affiliations:** Faculty of Dentistry, Institute of Oral Biology, University of Oslo, Oslo, Norway

**Keywords:** host, commensal, pathogen, lungs, vaccine

## Abstract

A diverse community of trillions of commensal bacteria inhabits mucosal and epidermal surfaces in humans and plays an important role in defense against pathogens, including respiratory pathogens. Commensal bacteria act on the host's immune system to induce protective responses that prevent colonization and invasion by pathogens. On the other hand, these bacteria can directly inhibit the growth of respiratory pathogens by producing antimicrobial products/signals and competing for nutrients and adhesion sites. Such mechanisms preserve the niche for commensal bacteria and support the host in containing respiratory infections. Herein, we discuss current evidence on the role of commensal bacteria in conferring protection against respiratory pathogens and the underlying mechanisms by which these bacteria do so. A deeper knowledge of how commensal bacteria interact with the host and pathogens might provide new insights that are poised to aid in the development of vaccines and therapeutics that target infectious diseases.

## Introduction

Since the inception of the Human Microbiome Project in 2007, a plethora of knowledge has accumulated that throws light on diverse and crucial roles played by commensal bacteria in homeostasis and disease (1, 2). With the help of advances in omic and systems biology technologies, our knowledge of the composition, genetics, and functional capacity of commensal bacteria is growing at a fast pace. It is becoming clear that commensal bacteria, which reside in various parts of the human body, such as the gut and airways, correspond approximately to the total number of human cells (about 1:1 ratio), and exert a profound impact on regulation of immunophysiological functions, including but not limited to, metabolism, ontogeny, and pathogen defense (3, 4). Several recent studies have shown that commensals promote resistance to gut pathogens that is mutually beneficial to the host and the commensal microbiota (5–7). However, imbalances in the microbial communities can occur, and are linked to many diseases, such as inflammatory bowel disease, allergies, asthma, diabetes, and obesity (8). It remains scantily understood how these bacteria execute their functional activities against respiratory pathogens. Respiratory infections are of utmost importance, as they inflict substantial social and economic burden on people across the world in general and in low and lower-middle income countries in particular (9–11). Additionally, current therapeutic and prophylactic interventions against respiratory diseases have major constraints, such as rapid emergence of anti-microbial resistance and disruption of the normal microbiota by use of antibiotics. Unraveling the interactions between commensals and pathogens may allow the exploitation of inhibitory properties of commensals to combat pathogens causing respiratory diseases. In this review article, we provide an overview of the current state of knowledge about the role of commensal bacteria in protective immunity to respiratory pathogens and the mechanisms involved in commensal bacteria mediated defenses. Understanding the relationship between commensal bacteria, host, and pathogen is a way forward to develop safe and effective prophylactics and therapeutics against pathogens.

## Commensal Bacteria Mediated Protection

### Protection in Mouse Models

Much of what is known about the direct role of commensal bacteria in protection against respiratory pathogens stems from studies using various mouse models, including germ-free and antibiotic-treated mice (12–23). Following lung infection with *Streptococcus pneumoniae*, numerous studies have shown that antibiotic-treated mice display significantly higher pathogen loads and increased pathologies in the lungs compared with sham-treated mice (15, 21). Likewise, germ-free mice showed enhanced levels of bacterial burden when subjected to *S. pneumoniae* and *Klebsiella pneumoniae* lung infections (15). Similar to the protection conferred by fecal microbiota transplant (FMT) against intestinal pathogens, FMT in gut microbiota-depleted mice restored pulmonary bacterial clearance early after *S. pneumoniae* infection (21). In case of mouse models of *Mycobacterium tuberculosis* infection, gut microbiota disruption after pre- and post-antibiotic treatment showed decreased resistance to infection in the lungs, which was associated with severe histopathological changes, such as pulmonary granulomas (24). Furthermore, antibiotic-induced dysbiosis changed the microbiota diversity in the gut and promoted lung colonization by *M. tuberculosis* (25). Similar protective effect was conferred after mice having antibiotic-induced disrupted microbiota received an intranasal infectious dose of influenza A virus (12). In a different study, FMT into germ-free mice led to reconstitution of the gut microbiota that facilitated increased survival against lethal influenza A virus infection (26). Overall, these studies employing multiple experimental approaches provide *in vivo* evidence that underscores a profound contribution of commensal bacteria in defense against diverse respiratory pathogens.

Even though antibiotic-treated and germ-free mice have proven to be a crucial tool in understanding the role of the microbiota in pathogen defense, there are potential pitfalls that need consideration while interpreting results from studies involving these animals. Germ-free animals lack all detectable microbes in different organs and have an impaired immune system, whereas antibiotics are used to deplete specific microbiota (27). Although these two approaches provide crucial information on the function of the microbiota in general, the specific contribution of the microbiota found in different body compartments, such as the lung microbiota, in immunity to respiratory infections is unclear. This is important because the lung microbiota, which in healthy adults seems to mainly consist of a small number of bacteria originating from the oral cavity, plays a significant role in respiratory health, and disease (28–30). Additionally, there is a need for models that can answer a more direct question about protection in the presence of a fully developed immune system. To address this issue, researchers have attempted to deplete the lung microbiota in mice by a combination of aerosolized vancomycin and neomycin via nasal route, which resulted in a significant reduction in the lung commensal microbiota, with the advantage of minimally affecting the gut microbiota (31, 32). But the possibility of antibiotic spread to the nearby tissues/organs harboring different microbiota remains, requiring future studies to focus on developing better models to fill in this pitfall.

The microbiota consists of a large number of bacterial species, and therefore, it is of great interest to specifically identify commensal species that protect from respiratory pathogens. Recent studies have evaluated the protective efficacy of commensal bacterial species in respiratory infections. Oral administration of *Bifidobacterium longum* (BB536), but not saline, in mice significantly reduced viral loads, pulmonary pathology, and body weight loss following intranasal challenge with influenza virus, suggesting a protective role for this commensal bacterium in influenza infection (33). Similarly, oral or nasal inoculation of mice with different strains of *Lactobacillus*, e.g., *L. gasseri* (TMC0356), *L. rhamnosus* (CRL 1505), and *L. brevis* (KB290), conferred protective immunity to influenza virus infections (34–36). Furthermore, *L. rhamnosus* (CRL 1505) exerted a protective effect in mice subjected to an intranasal challenge with respiratory syncytial virus infection (37, 38). These data indicate a prophylactic role for commensal bacteria against viral pathogens. In order to assess therapeutic significance, *B. longum* (MM2) was orally administered in mice infected with influenza virus. Mice that received *B. longum* (MM2) ameliorated infection, as determined by decreased body weight loss, viral titers, and inflammation, compared with control mice (39). Protective effect induced by these probiotic bacteria is not confined to respiratory infections with viruses, but can be applicable to bacterial pathogens (37–40). Intake of *B. longum* (5^1A^) in mice not only demonstrated protective effect against infection with *K*. *pneumoniae*, but also suppressed inflammatory changes in the lung (40). Very recently, we have demonstrated that intranasal immunization of mice with the commensal *Streptococcus mitis* conferred protection against lung infection with *S. pneumoniae* strains D39 (serotype 2) and TIGR4 (serotype 4), which illuminates the unique ability of *S. mitis* to offer resistance to different pneumococcal serotypes (41). Two recent studies performed by independent groups further show that the gut colonizer bacterium *Helicobacter hepaticus* influences the composition of the gut microbiota and the outcome of *M. tuberculosis* infection in mouse models (42, 43). Mice subjected to intestinal colonization with *H. hepaticus*, when challenged by intranasal route with *M. tuberculosis*, reflected higher mycobacterial burden in the lungs compared with the controls (42). This increased mycobacterial burden in the *H. hepaticus*-colonized mice coincided with severe *M. tuberculosis*-mediated pulmonary pathologies, mainly characterized by granulomas and tissue damage, and accumulation/production of inflammatory leukocytes/cytokines (42). Similar to these results, mice colonized with *H. hepaticus* eliminated subunit-vaccine-induced protective immunity to lung infection with *M. tuberculosis* (43). Taken together, these findings indicate that commensal bacteria can be harnessed for prophylactic and therapeutic purposes, provided utmost precaution on the possible negative effects of enriching for specific colonizers of the microbiota.

### Protection in Humans

Relatively little information is available on whether commensal bacteria can prevent respiratory infections in humans. Oral commensals, such as *Streptococcus oralis* and *Streptococcus salivarius*, can induce protection against middle ear inflammation, referred to as otitis media, which is primarily caused by respiratory pathogens, such as *S. pneumoniae* and *Haemophilus influenzae* (44–46). Upon intranasal administration of *S. salivarius* and *S. oralis*, children susceptible to acute otitis media displayed reduced recurrences of disease with no side effects (44). Contrarily, a nasal spray containing oral commensals, e.g., *S. mitis* and *S. oralis*, in susceptible children under 4 years of age showed no significant effect regarding the number of episodes of recurrent otitis media compared to the placebo group (46). The discrepancy in these studies might be due to differences in bacterial doses, inoculation regimens, and combinations, which need to be analyzed in light of new technologies (e.g., metagenomics and next generation sequencing) and concepts like dysbiosis. The fact that antibiotics were used together with the streptococcal nasal spray in the first study, but not in the second, is also an important factor to consider. In controlled infection studies in humans, nasopharyngeal colonization by the commensal *Neisseria lactamica* provided protection against the respiratory pathogen *Neisseria meningitidis* (47, 48). Furthermore, in a block-randomized challenge trial, 310 healthy individuals (18–25 years) were intranasally inoculated with live *N. lactamica* or sham and the bacterial carriage was monitored for 26 weeks (48). All those who developed nasopharyngeal colonization by *N. lactamica* revealed a significant reduction in the *N. meningitidis* carriage compared with sham-treated ones (48). These studies show that commensal bacteria not only show inhibitory/displacing effects on the carriage of respiratory pathogens but also highlight the ease and safety with which these bacteria can be used to contain infections in humans. It is however notable that most bacteria with high pathogenic potential, such as those in the above examples, are also part of the healthy microbiome (49). The reason as to why these pathogens cause diseases is attributed to various host and microbial factors, including viral infections (49). Dysbiosis in particular, such as a result of antibiotic use has been associated with a reduction in the prevalence of respiratory commensal bacteria like *Corynebacterium* spp. and *Dolosigranulum* spp. in the nasopharynx of healthy infants. These are considered to reduce the colonization by *S. pneumoniae, H. influenzae*, and *S. aureus* in the respiratory tract (50). It is further shown that respiratory syncytial virus infection in children below 2 years of age was positively correlated with nasopharyngeal *H. influenzae* and *Streptococcus* microbiota clusters and inversely correlated with *Staphylococcus aureus* (51). Transcriptomic analysis of the children infected with *H. influenzae* and *Streptococcus* clusters presented greater expression of immune components, suggesting that nasopharyngeal microbiota can influence host immunity (51). In line with this, prolonged antibiotic treatment in early life has also been annexed with an increased risk for respiratory infections in infants (52, 53). Thus, these studies shine light on the effect of the microbiota perturbations caused by antibiotics on host susceptibility to respiratory infections, particularly during the critical life period of immune maturation.

## Mechanisms of Commensal Bacteria Mediated Protection

A pertinent question however remains as to what are the underlying mechanisms by which commensal bacteria perform their protective function against respiratory pathogens. Emerging data thus far indicate that commensal bacteria confer protection in two ways: host-mediated immunity (acting on the host's immune system) and direct action (directly inhibiting/killing pathogens and competing for colonization).

### Host-Mediated Immunity

**Figure 1:**
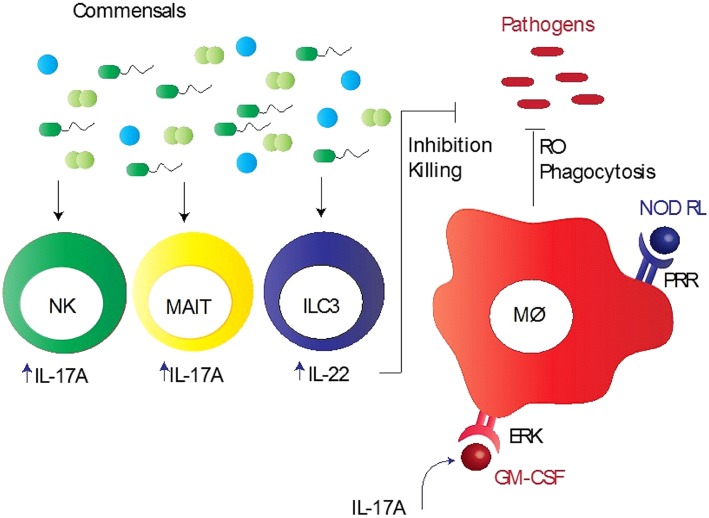
Commensal bacteria mediated innate immunity to respiratory pathogens. Commensal bacteria stimulate various innate immune cells, particularly alveolar macrophages (Mϕ), mucosa-associated invariant T (MAIT) cells, group 3 innate lymphoid cells (ILC3), and natural killer (NK) cells, to induce early protection. These bacteria promote pathogen killing via granulocyte–macrophage colony-stimulating factor (GM-CSF), which stimulates pathogen killing and clearance by alveolar macrophages (Mϕ) through phagocytosis, reduced reactive oxygen species (RO), and extracellular signal regulated kinase (ERK) signaling. Intrapulmonary GM-CSF production in response to infection is regulated by the microbiota via interleukin-17A (IL-17A). Pattern recognition receptor (PRR) expressed by Mϕ recognizes PRR ligands, such as nod-like receptor ligands (NOD RL), leading to the activation of Mϕ. NK and MAIT cells when activated by commensal bacteria produce large quantities of IL-17A, whereas ILC3 cells secrete IL-22, aiding in inhibition/killing of various respiratory pathogens.

A wealth of emerging evidence indicates that both the lung and gut microbiota are involved in the regulation of immune responses during lung infections (28, 54). However, it is difficult to assess the specific contributions of the lung and gut microbiota to protective immunity to respiratory pathogens, mainly due to three reasons: (1) the gut microbiota is the largest and most diverse community of commensals that significantly influences the outcome of immunity in the lungs as well as gut; (2) the gut is the largest lymphoid organ in the body because of which it occupies a central position in host-microbiota studies; and (3) we do not have optimal models to ascertain their specific roles in immunity. Despite an important role for these commensal bacteria in promoting resistance against respiratory pathogens, the mechanistic basis for this resistance remains unclear. Several studies have shown a potential defect in innate immunity and subsequent adaptive immunity in the lung, when signals from commensal bacteria are abrogated (12, 14, 25, 55). Key innate immune cells that are recruited to the lungs and are involved in protective immunity include macrophages, natural killer (NK) cells, and mucosal-associated invariant T (MAIT) cells (12, 14, 25, 55). The pulmonary macrophages in mice depleted of the microbiota by antibiotics reflected reduced expression of the macrophage-associated antiviral genes, such as Irf 7, Ifnb, Mx1, Tnfa, Il6, and Il1b following influenza virus infection (14). This corresponded with reduced response to IFN-γ, IFN-α, or influenza infection in macrophages from the mice treated with antibiotics. *In vivo* experiments in mice also indicated that the alveolar macrophage response was impaired during viral infection, which was characterized by down-regulation of most of the antiviral genes activated *ex vivo* (14). Wang et al. demonstrated a new mechanism in mice colonized with *S. aureus* where CD11b^+^ M2 alveolar macrophages, stimulated with Toll-like receptor (TLR) 2, play a protective role in influenza infection (56). Another innate immune cell type is MAIT cell that is shown to play an important role in microbiota mediated mycobacterial immunity (25). Flow cytometric analysis reflected that mice depleted of the microbiota had reduced number of lung MAIT cells, characterized by MR1-5-OP-RU tetramer^+^TCRβ^+^ phenotype, which expressed significantly lower IL-17A compared with control mice, suggesting that lung MAIT cells may function to contain early pulmonary *M. tuberculosis* infection (25). Furthermore, NK cells from germ-free mice did not induce anti-influenza immunity because macrophages and dendritic cells failed to produce type 1 IFN in response to infection (55). Cumulatively, these data suggest that microbiota-derived signals provide a stimulus that maintains the potency of the lung innate immune system needed for invoking effective immunity (Figure 1).

Adaptive immunity follows innate immunity and is crucial for specific immunity against respiratory pathogens (57–59). Rabbit antisera raised against *S. mitis* show cross-reactivity with *S. pneumoniae* (59). Similar to IgG mediated cross-reactivity, IgA antibodies from the sera, nasal wash, and bronchoalveolar lavage of mice vaccinated with *S. mitis* cross-reacted with *S. pneumoniae* serotypes 2 and 4 (41). On the other hand, human CD4^+^ T cells expressing IL-17A, which are reactive to *S. mitis*, show cross-reactivity toward *S. pneumoniae* in an *in vitro* culture system (60). Intranasal vaccination of mice with *S. mitis* led to an increased production of IL-17A by CD4^+^ T cells in the lungs compared to PBS-treated control mice (41). These results are in line with the evidence that the gut commensal segmented filamentous bacteria (SFB) regulate pulmonary Th17 immunity to the fungal pathogen *Aspergillus fumigatus* (19). In a mouse model of influenza viral infection, it is shown that commensal bacteria regulate virus-specific CD4^+^ and CD8^+^ T cells and antibodies following lung infection with virus (12). Overall, commensal bacteria mediated adaptive immunity to respiratory pathogens include both humoral (IgG and IgA) and T cell-mediated responses.

Involvement of the gut microbiota in protective immunity to pulmonary pathogens illustrates a pathogenic nexus between the microbiota and the “gut-lung axis,” underscoring a profound protective influence of the gut commensals over several pathogens residing at distant anatomical compartments of the body (61). The gut microbiota mediated control of the lung immunopathology is also evident from studies demonstrating the susceptibility of animals with the altered gut microbiota to allergic lung diseases (20). On the other hand, dysbiosis in the lung microbiota can change the composition of the gut microbiota. For example, mice subjected to lung infection with influenza virus infection or intranasal instillation of lipopolysaccharide disturbed the gut microbiota homeostasis, which supports the fact that the gut and lungs are closely linked in a way that they affect each other's microbiology and physiology (62, 63). Moreover, how the gut microbiota controls the lung immunity has recently been explored by few key studies in mouse models of pulmonary bacterial infections. Brown *et al*. performed a well-designed and comprehensive study that sheds light on a major innate immune mechanism used by the microbiota to clear lung infections in mouse models (15). In antibiotic-treated mice, there was an increased growth of *S. pneumoniae* and *K. pneumoniae* in the lungs after bacterial inoculation compared to sham-treated mice, which was associated with reduced production of innate immune factors, such granulocyte–macrophage colony-stimulating factor (GM-CSF) (15). *In vivo* neutralization of GM-CSF into antibiotic-treated mice, which received the microbiota from the sham-treated mice and had restored pulmonary bacterial clearance, resulted in making these mice prone to infections (15). These findings suggest that GM-CSF is essential for the microbiota to execute their functional activities against both Gram-positive (*S. pneumoniae*) and Gram-negative (*K. pneumoniae*) pathogens. It was further demonstrated that GM-CSF programs alveolar macrophage function via an extracellular signal-regulated kinase (ERK)-specific signaling pathway leading to increased pathogen killing via reactive oxygen species (ROS) (15). Several studies have implicated pattern recognition receptor (PRR) ligands produced by the gut microbiota in controlling immune responses outside the intestinal tract (16, 64). Following antibiotic-mediated depletion of the microbiota in mice, early clearance of *K. pneumoniae* was impaired and this could be rescued by injection of bacterial Nod-like receptor (NLR) ligands (the NOD1 ligand MurNAcTri(DAP) and NOD2 ligand muramyl dipeptide [MDP]), but not bacterial TLR ligands (16). Defects in early innate immunity were found to be due to reduced ROS-mediated killing of bacteria by alveolar macrophages (16). Interestingly, upon treatment of mice with antibiotics and NLR ligands orally prior to *S. pneumoniae* lung infection, neutralization of GM-CSF abrogated the rescue of respiratory clearance (15). Taking account of all these data, it is clear that the microbiota and NLR ligands regulate lung innate immunity to respiratory pathogens via GM-CSF, highlighting crucial mechanisms of the gut-lung axis of communication. In addition, the gut commensal SFB has been reported to provide protection in immunocompromised (Rag^−/−^) mice by partially enhancing neutrophil resolution during pneumococcal lung infection, which corresponded with reduced expression of the anti-phagocytic molecule CD47 (65). Like NK cells, another lymphoid cell population referred to as group 3 lymphoid cells that produce IL-22 (IL-22^+^ILC3), a cytokine involved in pathogen defense, has been implicated in gut commensal bacteria-induced protection against *S. pneumoniae* (66). Disruption of commensal bacteria by antibiotics decreased the influx of IL-22^+^ILC3 cells into the lungs of new born mice, which made them more prone to pneumococcal infection compared with control mice. This effect was reversed when ILC3 cells were adoptively transferred or exogenous IL-22 administered in mice (66). Thus, these immune mechanisms furnish crucial information on how the gut microbiota controls protective immunity to lung infections (Figure 1).

### Direct Action

Commensal bacteria resist colonization of pathogens by using wide range of direct mechanisms for niche competition, such as secretion of inhibitory substances and nutrient competition, enlisting the exploitative, and interference modes of competition (67). Recent studies dissected novel mechanisms used by resident commensals to inhibit and contain respiratory pathogens, such as disruption of biofilms, exploitation of host resources to generate antimicrobial products, and down-regulation of virulence genes. This highlights the complexity and diversity of mechanisms involved in direct inhibition (68–70). The well-documented mechanism by which commensal bacteria can directly inhibit the pathogen growth and compete with them is the production of ribosomally produced antimicrobials called bacteriocins (71, 72). For example, *S. salivarius* produces a wide range of bacteriocins, which is a major mechanism that antagonizes *S. pneumoniae* (71, 73–75). More recently, it is also demonstrated that *S. salivarius* reduces the *S. pneumoniae* colonization by blocking the adhesion sites, suggesting multiple mechanisms used by this commensal to inhibit pathogens (76). Apart from ribosomally encoded bacteriocins, commensal bacteria encode non-ribosomally produced bioactive antimicrobials to compete with pathogens (77). Zipperer et al. showed that the nasal commensal *Staphylococcus lugdunensis* directly inhibits the growth of *S*. *aureus* through a novel cyclic antimicrobial peptide named “Lugdunin.” Lugdunin possessed bactericidal activity against all tested strains of *S. aureus* in *vitro*. Moreover, in animal model, the co-colonization of *S. lugdunensis* and *S. aureus* resulted in competitive exclusion of *S. aureus* (77). The use of purified antimicrobials or bacterial strains encoding antimicrobials may serve as a source of new generation of antibiotics to deal with multidrug resistant strains, such as methicillin resistant *S. aureus*. One mechanism, which contributes to competitive advantage for colonization of commensal bacteria to preserve their niche and to suppress the growth of pathogens, includes the production of hydrogen peroxide. Epidemiological data show a negative correlation between *S. pneumoniae* and *S. aureus* and presumably, the reason for increased *S. aureus* related otitis media after use of pneumococcal vaccine (78, 79). One possible mechanism implicated to define this negative association is hydrogen peroxide mediated inhibition of *S. aureus* by pneumococcal hydrogen peroxide (80).

Nutrient competition is also a strategy used by commensal bacteria to reduce the fitness of pathogens by competing for the same pool of resources (81). Stubbendieck et al. recently showed that isolates of *Corynebacterium* spp. inhibited *Staphylococcus* spp. *in vitro*. This inhibition was due to reduced iron bioavailability, mediated by siderophore–induced sequestration of iron by *Corynebacterium* spp. (82). Another novel mechanism of commensal mediated inhibition is through the production of secreted enzymes. Iwase et al. first demonstrated the negative correlation between the commensal *Staphylococcus epidermidis* and pathogenic *S. aureus* in human nasal samples. To gain further insight to explain this negative association, they identified the inhibitory factor produced by *S. epidermidis* as serine protease, which inhibits the biofilm formation and human nasal colonization by *S. aureus* (68). Follow-up study from the same group showed that intranasal colonization of mice with serine protease producing *S. epidermidis* inhibited colonization with methicillin resistant *S. aureus* (83). Commensal bacteria also exploit the host resources to generate metabolic compounds with antimicrobial properties to suppress the growth of respiratory pathogens. An elegant study by Bomar et al. investigated the mechanistic explanation for correlation between increased abundance of *Corynebacterium* species and reduced *S. pneumoniae* colonization (69). Interestingly, they found that *Corynebacterium accolens* encodes lipase, which catalyzes the hydrolysis of host triacylglycerolsto to produce free fatty acids with antibacterial properties that suppress the growth of *S. pneumoniae* (69). Taken together, the above examples evidently suggest that antagonistic interactions exist in the polymicrobial community utilizing wide range of mechanisms by which commensal bacteria inhibit respiratory pathogens. Advanced understanding of existing mechanisms using both *in vitro* and *in vivo* models and further elucidation of novel mechanisms may enable us to exploit commensals to inhibit respiratory pathogens. Mechanisms used by commensal bacteria to directly inhibit/kill respiratory pathogens are exemplified in Table 1.

**Table 1:** Examples of direct mechanisms of colonization resistance used by commensal bacteria against respiratory pathogens.

**Commensal bacteria**	**Anatomical location**	**Mechanism of inhibition**	**Respiratory pathogens**	**References**
*Streptococcus salivarius*	Oral cavity	Ribosomally synthesized antimicrobials (Bacteriocins)	*Streptococcus pneumoniae Streptococcus pyogenes*	(71)
*Staphylococcus lugdunensis*	Skin, and nasal cavity	Non-ribosomally synthesized antimicrobials (Lugdunin)	*Staphylococcus aureus*	(77)
*Corynebacterium accolens*	Skin, and nasal cavity	Metabolic products with antimicrobial properties (Free fatty acids)	*Streptococcus pneumoniae*	(69)
*Staphylococcus epidermidis*	Skin and nasal cavity	Secreted enzymes (Serine protease)	*Staphylococcus aureus*	(68)
*Streptococcus pneumoniae*	Nasopharynx, and oral cavity	Hydrogen peroxide (H_2_0_2_) mediated killing	*Staphylococcus aureus*	(80)
*Corynebacterium* spp.	Skin and nasal cavity	Nutrient competition (Iron limitation by siderophore production)	*Staphylococcus* spp.	(82)

## Conclusions and Future Insights

Advanced research technologies have been applied to evaluate the contribution of commensal bacteria to respiratory infections. Accumulating evidence indicates an important role for commensal bacteria in defense against respiratory pathogens, which paves the way to target these bacteria for the development of vaccines and therapeutics that provide optimal protection with safety and low cost. Moreover, the use of modern experimental tools to decipher the novel mechanisms used by commensals to inhibit pathogens may assist in designing novel therapeutics with targeted approach focusing exclusively on the pathogen inhibition without disrupting the homeostatic microbial community. Future studies are required to address the following questions: (1) What are the underlying mechanisms by which the trio of commensals, pathogens, and host interact with each other? (2) What could be the long-term consequences of using commensal bacteria-based vaccines/therapeutics on the host, pathogens, and the microbiota? (3) What are the effects of medical manipulations, such as antibiotics and probiotics, on the biology of commensal bacteria? (4) How can we use commensal bacteria-expressed bacteriocins for protection against respiratory pathogens? (5) Which specific commensal bacterial species of the microbiota are directly involved in protection immunity to different pathogens? (6) How can we use commensal microbiota/bacteria to correct dysbiosis? A sincere exploration of these questions may have implications for the clinical use of commensal bacteria with inhibitory properties against pathogens. This may be important to bypass the drawbacks associated with currently available options, such as antimicrobial resistance.

## Author Contributions

RK, FP, and SS wrote and revised the manuscript. All authors assisted in the conception of this review and acquisition of relevant literature. All authors gave approval of the last version to be published.

### Conflict of Interest Statement

The authors declare that this work was conducted in the absence of any commercial or financial relationships that could be construed as a potential conflict of interest.
